# Establishment of a Cell-Fusing Agent Virus Infection Model in *Aedes albopictus* and Its Impact on Vector Competence for Zika Virus

**DOI:** 10.3390/v18030384

**Published:** 2026-03-19

**Authors:** Dongqin Li, Ningxin Zhou, Li Xiong, Xi Pu, Mingqiang Li, Qing Liu, Lu Liu, Rui Xiao, Yuanhang Wang, Hengduan Zhang, Xiaoxia Guo, Dan Xing, Tongyan Zhao, Jiahong Wu, Yuting Jiang

**Affiliations:** 1 The Key Laboratory of Environmental Pollution Monitoring and Disease Control, Ministry of Education, School of Public Health, Guizhou Medical University, Guiyang 561113, China; 18722955163@163.com (D.L.); x1095456@163.com (L.X.); 2 State Key Laboratory of Pathogen and Biosecurity, Academy of Military Medical Sciences, Beijing 100071, China; 15755498580@163.com (N.Z.); puxi0080@163.com (X.P.); limq0307@163.com (M.L.); 15290116829@163.com (Q.L.); l3l27u@163.com (L.L.); 18085898122@163.com (R.X.); 18639004072@163.com (Y.W.); zhanghengduan1987@163.com (H.Z.); guoxx99@163.com (X.G.); xingdan93@163.com (D.X.); tongyanzhao@126.com (T.Z.); 3 Characteristic Key Laboratory of Modern Pathogen Biology, School of Basic Medicine Sciences, Guizhou Medical University, Guian New Area 561113, China

**Keywords:** Cell-fusing agent virus (CFAV), *Aedes albopictus*, insect-specific virus (ISV), vector competence, Zika virus (ZIKV)

## Abstract

The overuse of chemical insecticides highlights the urgent need for novel vector control strategies. Insect-specific viruses (ISVs), such as the cell-fusing agent virus (CFAV), have shown potential to block arbovirus transmission by inhibiting viral replication in mosquitoes. However, the effects of CFAV beyond its natural host, *Aedes aegypti*, remain largely unexplored. In this study, we established a CFAV infection model in *Aedes albopictus*, a major vector for Zika virus (ZIKV), via intrathoracic injection. Stable infection was achieved, with viral loads reaching up to 10^7^ copies per mosquito by day 10 post-injection. Nevertheless, high post-injection mortality (median survival: 3 days) was observed, which we attribute primarily to mechanical injury. No evidence of vertical transmission of CFAV was detected in *Ae. albopictus*. Co-injection of CFAV and ZIKV did not significantly affect ZIKV replication in this species. In contrast, in *Ae. aegypti* pre-infected with CFAV followed by oral ZIKV challenge, CFAV significantly reduced ZIKV infection rates in the ovaries at day 4 and viral loads in salivary glands at day 10. These findings demonstrate that while CFAV can productively infect *Ae. albopictus*, it does not undergo vertical transmission in this species, and has no inhibitory effect on ZIKV under the co-infection conditions tested. This study underscores challenges associated with using single ISVs such as CFAV for arbovirus control and highlights the complex, bidirectional role of multiple ISV co-infections. While exploring multi-ISV combinations may offer a potential strategy to enhance antiviral efficacy, their net effect—whether suppression or enhancement of arboviruses—warrants careful investigation.

## 1. Introduction

*Aedes albopictus* is an important vector for mosquito-borne infectious diseases such as Zika virus disease, dengue fever, and chikungunya fever, posing a serious threat to public health [1]. To mitigate this threat, insecticides are frequently employed. However, the overuse of insecticides not only leads to the development of insecticide resistance in mosquitoes but may also cause severe damage to the environment and ecosystems [2]. Consequently, the scientific community continues to explore and develop novel, effective, and environmentally friendly strategies for controlling vector-borne diseases. Among these, approaches based on mosquito intracellular symbiotic microorganisms to suppress arboviruses represent a highly promising approach for blocking disease transmission.

The most common intracellular symbiotic microorganisms in mosquitoes include *Wolbachia* and insect-specific viruses (ISVs). *Wolbachia*, a rickettsial bacterium found in terrestrial arthropods and filarial nematodes [3], has been extensively studied for its ability to confer against various arboviruses, with systematic reviews highlighting the differential effects of *Wolbachia* strains carried by *Ae. Albopictus* and *Aedes aegypti* [4]. ISVs are a group of viruses that infect only insects and do not replicate vertebrates or vertebrate cells [5]. With advancement in high-throughput sequencing and intensified mosquito surveillance, an increasing number of ISVs have been detected or isolated from mosquitoes. Several ISVs, including Cell-fusing agent virus (CFAV) [6], Palm Creek virus (PCV) [7], Nhumirim virus [8,9], and Espirito Santo virus [10], have been demonstrated to inhibit arbovirus replication in mosquitoes or reduce viral transmission.

Introducing a novel intracellular symbiotic microorganism into mosquitoes that do not naturally carry it often leads to more effective resistance against arboviruses. This effect may arise from two mechanisms: first, the newly introduced microorganisms can activate the mosquito’s immune pathways, thereby enhancing its ability to suppress arboviruses [11]; second, it may induce superinfection exclusion, whereby prior infection with a homologous virus (and in some cases, closely related viruses) competitively suppresses the replication of a superinfecting virus [5,12]. CFAV was the first ISV to be discovered [13], and several studies have demonstrated its antiviral effects against specific arboviruses in vitro [14] or in vivo [6], highlighting its potential for use in strategies aimed at controlling vector-borne diseases. While CFAV has since been documented in natural populations of both *Ae. aegypti* and *Ae. albopictus* [15], experimental evidence for its antiviral or superinfection exclusion effects has been predominantly established in *Ae. aegypti* [6,14]. The capacity of CFAV to infect and stably colonize *Ae. albopictus*, and crucially, whether it can exert a similar arbovirus-blocking effect in this species, remains largely unexplored. Therefore, this study aims to investigate the infectivity of CFAV in *Ae. albopictus* and to explore the feasibility of using a CFAV-infected *Ae. albopictus* model to block arbovirus transmission.

## 2. Materials and Methods

### 2.1. Mosquitoes

The *Ae. Albopictus* (Guangzhou strain) was originally from field collections in Guangzhou City, Guangdong Province (GPS location: 23°07′ N and 113°16′ E) during 2019. *Aedes aegypti* (Menghai strain) was obtained from Menghai County in Yunnan Province, China (GPS location:21°57′ N, 100°27′ E) in the same year. All mosquitoes were reared under controlled insectary settings: temperature 26 ± 1 °C, relative humidity 75 ± 5%, and a photoperiod cycle of 14 h light and 10 h darkness. Adults were provided with 8% sucrose solution ad libitum. As previously reported, the *Ae. aegypti* strain was confirmed to be CFAV-free via RT-qPCR and cell culture assays [16]. Similarly, the *Ae. albopictus* colony used in this study was tested by RT-qPCR and found negative for CFAV RNA.

### 2.2. Viruses

CFAV was obtained from the Aag2 cell lines as previously reported [16]. The virus was amplified in *Ae. albopictus* C6/36 cells (ATCC Number: CRL-1660), yielding a working stock with a concentration of 3.3 × 10^7^ RNA copies/μL. ZIKV SZ01 strain used in this study was originally isolated from a patient who returned from Samoa to China in 2016 [17] (GenBank accession number: KU866423). The virus has been passaged in the C6/36 cell lines 9 times. The ZIKV stock had a titer of 6 × 10^7^ plaque-forming units (PFU)/mL, corresponding to 1.8 × 10^8^ RNA copies/μL.

### 2.3. Intrathoracic Injection

Microinjection into the mosquito thorax was conducted as reported previously [16]. In brief, 7-day-old female *Ae. albopictus* were anaesthetized with CO_2_ and placed on a cooling plate. Using a FemtoJet 4i microinjector (Eppendorf, Hamburg, Germany), 300 nL of virus preparation or phosphate-buffered saline (PBS) was delivered intrathoracically. Post-injection, mosquitoes were reared in standard insectary and the survival was monitored.

### 2.4. Examination of Vertical Transmission (VT) of CFAV in Ae. albopictus

A cohort of 200 CFAV-inoculated parental (F0) female *Ae. albopictus* were allowed to blood-feed on Kunming mice (Beijing Vital River Laboratory Animal Technology, Beijing, China) for propagation on days 3, 10 and 17 post-infection (dpi). The mosquitoes were provided with filter paper placed in a small container with water for oviposition on 6–9 dpi (the first gonotrophic cycle, GC1), 13–16 dpi (GC2) and 20–23 dpi (GC3). Eggs from each cycle were harvested, hatched, and the resulting F1 progeny were screened for CFAV infection at 3 days after emergence.

### 2.5. Coinfection of CFAV and ZIKV in Ae. albopictus

For coinfection studies, ZIKV was diluted 5.5-fold with 1640 medium to achieve the same copy concentration as CFAV (3.3 × 10^7^ RNA copies/μL). The two viruses were then mixed in equal volumes to infect *Ae. albopictus* via intrathoracic injection. Mosquitoes in the control group were injected with a mixture of diluted ZIKV preparation and 1640 medium. Whole mosquitoes were collected for total RNA extraction at 0, 1, 2, 3, 4, 6, 8, and 10 days post-infection for ZIKV RNA detection.

### 2.6. ZIKV Superinfection After CFAV Injection in Ae. aegypti

Seven-day-old adult female *Ae. aegypti* was first injected with CFAV. Two days later, these mosquitoes were subjected to an infectious blood meal containing ZIKV. ZIKV-infected blood meals were prepared by mixing 1:1 mouse blood and ZIKV suspension supplemented with 2% FBS and 1% heparin sodium. After 18 h of starvation, CFAV-injected mosquitoes were fed using a Hemotek membrane feeder maintained at 37 °C. Engorged females were selected after 1 h of feeding, and blood-engorged mosquitoes were transferred to and maintained in the standard rearing conditions.

### 2.7. Mosquito Processing and RNA Extraction

For tissue-specific ZIKV detection in *Ae. aegypti*, the mosquitoes were dissected with sterile dissecting needles, then the salivary gland, midgut, and ovary were collected individually and transferred into 1.5 mL microtubes containing 1 mL of RNAiso Plus (TaKaRa, Dalian, China). Total RNA was then extracted according to the manufacturer’s instructions. For CFAV or ZIKV detection in *Ae. albopictus*, the whole body was directly used for RNA extraction without prior dissection.

### 2.8. RT-qPCR Detection

Virus and endogenous reference gene transcripts were quantified by the GoTaq Probe 1-Step RT-qPCR System (Promega, Madison, WI, USA). Each 20 μL reaction contained: 10 μL of GoTaq Probe qPCR Master Mix, 0.4 μL of GoScript RT Mix, 1 μL each of forward primer, reverse primer, and probe for virus, 1 μL each of forward primer, reverse primer, and probe for host reference gene, 2 μL of RNA template, and 1.6 μL of nuclease-free water. Amplification was carried out on a QuantStudio 7 Flex instrument (Thermo Fisher Scientific, Waltham, MA USA) under the following conditions: 1 cycle at 45 °C for 15 min, 95 °C for 10 min, 40 cycles at 95 °C for 15 s and 60 °C for 30 s. Absolute quantification was achieved using standard curves generated from serial dilutions of relevant recombinant plasmids. Primer and probe sequences are provided in Table 1.

### 2.9. Statistical Analysis

Statistical analyses and graphing were performed with GraphPad Prism software (version 9.4). Survival distributions between PBS- and CFAV-injected *Ae. albopictus* groups were compared via Kaplan–Meier analysis with the log-rank (Mantel–Cox) test. Residual normality and homoscedasticity were verified using the Shapiro–Wilk test and Spearman’s correlation test, respectively. Temporal changes in CFAV load within *Ae. albopictus* were analyzed by one-way ANOVA with Tukey’s multiple comparisons test. Comparisons of ZIKV loads over time between infection groups (CFAV vs. medium) were made using two-way ANOVA followed by Šídák’s multiple comparisons test. Infection rates were compared using Pearson’s chi-square test. A *p*-value below 0.05 was deemed statistically significant.

## 3. Results

### 3.1. CFAV Can Infect and Replicate in Ae. albopictus

**Figure 1:**
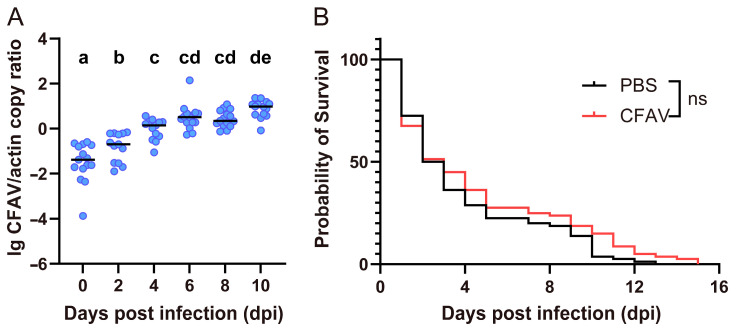
Replication kinetics of CFAV in and survival curve of *Aedes albopictus* after intrathoracic injection. (**A**) Female *Ae. albopictus* was infected with CFAV via intrathoracic injection. The viral RNA and actin mRNA on the indicated days were examined by RT-qPCR (*n* = 15). The CFAV load was normalized to the host actin mRNA and the results were expressed on a log10 scale. Blue dots represent the CFAV load and the mean values are indicated by the horizontal line for each group. Letters above the graphs represent the statistical significance of pairwise differences after one-way ANOVA with Tukey’s multiple comparisons test. The differences between groups with a letter in common are not statistically significantly different. (**B**) Survival curve of *Ae. albopictus* after intrathoracic injection. Female *Ae. albopictus* mosquitoes were intrathoracically injected with CFAV or phosphate-buffered saline (PBS) (*n* = 80 in each group) and the survival of mosquitoes within 15 days was monitored. Survival curves of the two groups were compared by Kaplan–Meier survival analysis with a log-rank (Mantel–Cox) test. ns No significant difference.

To determine whether CFAV can infect *Ae. albopictus*, the virus was introduced into the mosquitoes via intrathoracic injection, and viral replication was monitored over a 10-day period. As shown in Figure 1A, CFAV viral load increased progressively over time, with the mean load (expressed as the log_10_-transformed ratio of CFAV to actin copy numbers) rising from −1.50 on day 0 to 0.87 on day 10. The viral load on day 0 was significantly lower than that at all subsequent time points, while the load on day 10 was significantly higher than that on day 4 and earlier time points. Notably, *Ae. albopictus* appeared to be highly susceptible to the physical damage caused by intrathoracic injection. Following CFAV injection, the median survival of *Ae. albopictus* was only 3 days, which is significantly shorter than the 13 days observed in our previous study with *Ae. aegypti* [16]. However, no significant difference in survival was observed between *Ae. albopictus* injected with CFAV and those injected with PBS (Figure 1B). This indicates that CFAV itself does not affect the survival of *Ae. albopictus*, and that the reduced survival time is attributable to mechanical damage resulting from the injection procedure.

### 3.2. CFAV Is Not Vertically Transmitted in Ae. albopictus

Our previous research demonstrated that *Ae. aegypti* artificially infected with CFAV do not transmit the virus to their offspring [16]. The present study aimed to investigate whether this phenomenon also occurs in *Ae. albopictus*. Parental generation *Ae. albopictus* (F0) were injected with CFAV and allowed to lay eggs over three subsequent GCs. The eggs were hatched, and on the third day after emergence, a specified number of F1 offspring mosquitoes were randomly selected for CFAV detection. However, no CFAV was detected in any of the tested individuals, resulting in an infection rate of 0% (Table 2). These results indicate that, similar to *Ae*. *aegypti*, *Ae. albopictus* artificially infected with CFAV also do not transmit the virus to their progeny.

### 3.3. CFAV Does Not Affect ZIKV Replication in Ae. albopictus

**Figure 2:**
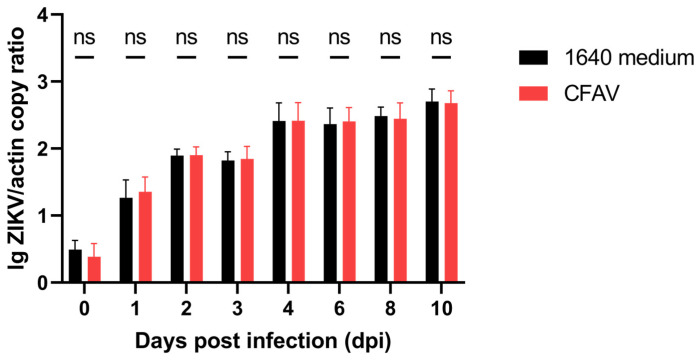
Replication kinetics of ZIKV in the *Ae. albopictus* after coinfection of ZIKV and CFAV. *Ae. albopictus* mosquitoes were injected intrathoracically with a mixture of CFAV and ZIKV, while the control group was injected with a mixture of 1640 medium and ZIKV. ZIKV replication within the mosquitoes was detected by RT-qPCR over 10 days (*n* = 10 in the 1640 medium group at 10 dpi, and *n* = 15 in other groups). ZIKV load was normalized to the host actin mRNA and the results were expressed on a log10 scale. Data were presented as mean ± SD, and analyzed using two-way ANOVA with Šídák’s multiple comparisons test. Due to the high mortality rate in *Ae. albopictus* following intrathoracic injection, the presented results were combined from two independent trials. ns: No significant difference.

We next sought to determine whether co-infection with CFAV alters the vector competence of *Ae. albopictus* for ZIKV. We initially attempted to inject CFAV first, followed by oral infection with ZIKV. However, a substantial proportion of the mosquitoes died following intrathoracic injection, and the blood-feeding rate among survivors was low, making it difficult to obtain a sufficient number of engorged individuals for subsequent ZIKV detection. Therefore, in this study, we opted to co-inject CFAV and ZIKV simultaneously to investigate their interactions within the host. The control group received a mixture of ZIKV and an equivalent volume of 1640 medium in place of CFAV. ZIKV replication was then monitored over a 10-day period. As shown in Figure 2, ZIKV was detectable immediately after injection (day 0). No significant difference in viral load was observed between the two groups at this time point, confirming that the mosquitoes in both groups received comparable initial doses of ZIKV. From 1 dpi onward, ZIKV loads increased progressively in both groups. However, at no time point was there a statistically significant difference in ZIKV load between the CFAV-co-injected group and the 1640 medium control group. These results indicate that, over the 10-day observation period, CFAV does not affect ZIKV replication in *Ae. albopictus*.

### 3.4. CFAV Reduces Vector Competence of Ae. aegypti for ZIKV

**Figure 3:**
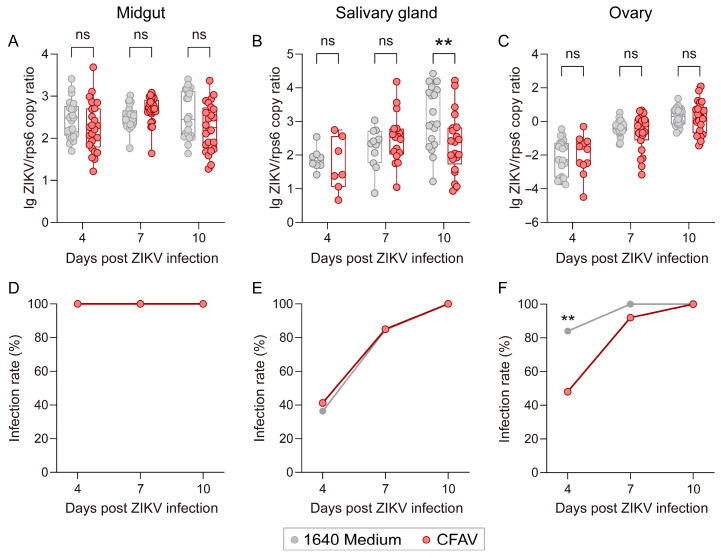
ZIKV replication in different tissues of *Aedes aegypti* pre-injected with CFAV or 1640 medium. (**A**–**C**) ZIKV load in the midgut, salivary gland, and ovary on different days-post infection. Box plots show the median and the 25th to 75th percentiles, and the whiskers denote the minimum and maximum values. Data were analyzed using two-way ANOVA with Šídák’s multiple comparisons test. (**D**–**F**) ZIKV infection rate in the midgut, salivary gland, and ovary on different days-post infection. Infection rate between two groups were compared by Pearson’s chi-square test. ** *p* < 0.01, ns: no significant difference.

Given that *Ae. aegypti* exhibits greater tolerance to intrathoracic injection, we selected this species to further investigate the effect of CFAV pre-infection on subsequent oral ZIKV challenge. Female *Ae. aegypti* were injected with a CFAV suspension, while control mosquitoes received an equal volume of 1640 medium. Two days post-injection, mosquitoes were orally exposed to ZIKV. At 4, 7, and 10 days post-exposure, ZIKV loads were measured in the midgut, salivary gland, and ovary. As shown in Figure 3, no significant differences in ZIKV loads or infection rates were observed in the midgut between the CFAV-pre-infected and control groups at any of the time points examined (Figure 3A,D). In the salivary gland, however, a significant lower ZIKV load was detected in the CFAV-pre-infected group at day 10 post-exposure (*p* = 0.0076), despite no significant difference in infection rates between the groups (Figure 3B,E). In the ovary, while viral loads did not differ significantly between groups at any time point (Figure 3C), the infection rate was significantly reduced in the CFAV-pre-infected group at day 4 post-exposure (χ^2^ = 7.219, df = 1, *p* = 0.0072, Figure 3F). Taken together, these results indicate that CFAV pre-infection can reduce the vector competence of *Ae. aegypti* for ZIKV, with tissue- and time-specific effects.

## 4. Discussion

This study aimed to introduce CFAV into *Ae. albopictus*, a major vector for arboviruses, in order to investigate the replication of an ISV in this species and to assess its impact on host survival and vector competence. Following intrathoracic injection, CFAV replicated stably in *Ae. albopictus*, reaching an average viral load of 10^7^ copies per mosquito by day 10 post-injection (with a normalized value of 0.87, Figure 1A). This level of replication was comparable to that previously observed in artificially infected *Ae. aegypti* [16], indicating that CFAV is capable of successfully infecting multiple mosquito species within the genus *Aedes*. However, the natural distribution of CFAV appears to be mainly associated with *Ae. aegypti* populations in the wild [18,19]. This host restriction may reflect the reliance of CFAV on VT as its primary maintenance mechanism in this species.

Our experiments revealed a notable phenomenon: *Ae. albopictus* exhibited unusually high mortality following intrathoracic injection. In this study, the median survival time of *Ae. albopictus* injected with CFAV was only three days. Similar high mortality rates were also observed in our other studies involving injection of dsRNA, siRNA, or water. This issue substantially increased the difficulty and workload of experiments involving *Ae. albopictus*, necessitating the use of larger initial sample sizes to obtain sufficient valid data. No significant difference in survival was observed between *Ae. albopictus* injected with CFAV and those injected with PBS, indicating that the high mortality was attributable to mechanical damage from the injection procedure rather than to CFAV itself. In contrast, other mosquito species, such as *Ae. aegypti*, demonstrated greater tolerance to injection-induced injury in our previous studies, with a median survival time of 13 days following CFAV or PBS injection [16]. At present, the underlying cause of this species-specific susceptibility remains unknown. Future research could explore alternative delivery methods, such as, ingestion, oral feeding, and lipid- or nanoparticle-coated delivery, which have been successfully applicated for dsRNA delivery in mosquitoes [20], as potential replacements for intrathoracic injection to improve experimental efficiency.

This study further investigated whether artificial infection with CFAV could lead to VT in *Ae. albopictus*. The parental generation of *Ae. albopictus*, injected with CFAV, laid eggs over three consecutive GCs. Viral RNA was undetectable in any offspring from the three GCs, regardless of sex (Table 2). Our preliminary studies also showed that CFAV is not vertically transmitted via artificial infection in *Ae. aegypti* [16]. A striking discrepancy exists in the VT rates (VTRs) of CFAV between naturally and artificially infected mosquitoes. In naturally infected *Ae. aegypti* populations, CFAV exhibits high VTR, whereas under artificial infection conditions, VT is extremely rare for both CFAV and other orthoflaviviruses such as ZIKV. Nag et al. recently provided a key anatomical observation: in naturally infected mosquitoes, CFAV distributes throughout the ovarian follicles and germaria after a blood meal [21], whereas in artificially infected mosquitoes, the virus is largely confined to the oviducts [22]. This suggests that natural infection enables transovarial transmission, i.e., direct germline infection, whereas artificial infection likely results in transovum transmission, where the virus contaminates the egg surface during oviposition. These observations imply the existence of a physical or immunological barrier surrounding the germinal cells that prevents viral entry into the ovarioles. However, how CFAV initially breached this barrier remains unclear; possible scenarios include sporadic transovum transmission events followed by adaptation, or the barrier evolving as a host defense after ancestral viral integration.

One of the key medical interests in CFAV lies in its potential to inhibit the transmission of pathogenic arboviruses. In this study, we evaluated the impact of CFAV on ZIKV replication in both *Ae. albopictus* and *Ae. aegypti*. Due to high post-injection mortality and low blood-feeding rates, we were unable to perform oral infection and instead adopted a co-injection strategy, simultaneously introducing CFAV and ZIKV into *Ae. albopictus*. Under this infection route, ZIKV bypassed the midgut infection barrier and replicated rapidly, reaching relatively high levels by day 4 post-infection. At this time point, the ZIKV loads (normalized to actin) in the CFAV-infected group and the control group were 2.42 and 2.41, respectively. By day 10, the levels reached 2.68 and 2.70 (Figure 2). These values were nearly two orders of magnitude higher than the peak CFAV load observed in *Ae. albopictus* (0.87, Figure 1A). However, at all tested time points, no statistically significant difference in ZIKV load were observed between the two groups, indicating that CFAV does not affect the overall replication capacity of ZIKV in *Ae. albopictus*. To better simulate the natural dissemination process of ZIKV from the midgut to other tissues following oral infection in the context of prior CFAV infection, we conducted superinfection experiments in *Ae. aegypti.* CFAV was injected into the thorax of *Ae. aegypti* two days before oral infection with ZIKV. ZIKV RNA levels were subsequently measured in the midgut, salivary gland, and ovary at specific time points. The results showed that, compared to the control group, the CFAV-preexposed group exhibited a significantly lower ZIKV infection rate in the ovary on day 4 and a reduced viral load in the salivary gland on day 10. The limited inhibitory effect of CFAV on ZIKV replication observed in this study may be attributed to two factors. First, peak CFAV loads were approximately two orders of magnitude lower than those of ZIKV in both mosquito species, which could limit the ability of CFAV to effectively compete for host resources or trigger sustained antiviral responses [23]. Second, the experimental designs may not have allowed sufficient time for CFAV to establish infection prior to ZIKV challenge: in *Ae. albopictus*, the two viruses were co-injected simultaneously, while in *Ae. aegypti*, the interval between CFAV injection and oral ZIKV challenge was only two days. These temporal constraints may have prevented CFAV from reaching the replication levels or tissue distribution required for effective interference.

Several methodological limitations should be considered when interpreting the findings of this study. First, due to the high post-injection mortality and low blood-feeding rates observed in *Ae. albopictus*, we were unable to perform oral infection with ZIKV and instead employed an intrathoracic co-injection model to assess viral interference. While this approach ensures synchronous and systemic delivery of both viruses, it bypasses the natural midgut infection and escape barriers—critical anatomical checkpoints that arboviruses must overcome during oral infection. Consequently, the co-injection model may not fully recapitulate the complex dynamics of superinfection under natural conditions, where tissue-specific barriers and temporal delays in viral dissemination could influence the outcome of ISV-arbovirus interactions. Second, the artificial infection route and the use of injected viral doses that differ from those acquired naturally may also affect viral replication kinetics and host immune responses, potentially masking or attenuating inhibitory effects that would otherwise occur in the field. Third, the observed mortality and reduced engorgement rates in injected mosquitoes raise the possibility that only a subset of more robust individuals were included in the analysis, which could introduce selection bias. Fourth, our assessment of VT was based solely on testing adult progeny for the presence of CFAV, rather than examining larval stages. This approach may have failed to account for potential transstadial loss of infection, where infected larvae could lose the virus during development, leading to an underestimation of true VTRs. Future studies incorporating pooled egg or larval samples to determine the minimum filial infection rate would provide a more robust assessment of vertical transmission and help clarify whether CFAV undergoes transstadial loss in *Ae. albopictus*. Additionally, exploring alternative delivery methods—such as oral infection or nanoparticle-based approaches—may offer viable replacements for intrathoracic injection, thereby enhancing the feasibility and ecological relevance of ISV-arbovirus interaction studies.

Despite their promising potential, a considerable gap remains between the current understanding of CFAV and other ISVs and their practical application in controlling arboviral diseases. First, ISVs—particularly those belonging to the orthoflavivirus group, including CFAV—are difficult to maintain stably alongside mosquito reproduction through artificial infection. Second, the antiviral effect of CFAV is limited to specific time points and particular tissues, and the resulting reduction in infection rate or viral load is moderate and insufficient to completely block viral replication. These limitations highlight the need for more sophisticated strategies to harness ISVs for arbovirus control. One promising avenue involves the strategic use of multi-ISV co-infections to achieve additive or synergistic antiviral effects. Field metagenomic studies have revealed that wild mosquitoes often harbor complex viromes consisting of multiple ISVs from diverse families, including flaviviruses, alphaviruses, and negeviruses [24,25,26], suggesting that certain ISV combinations may be compatible and could collectively modulate arbovirus replication—either through suppression or enhancement—depending on the specific virus combination and ecological context. For instance, co-infection with multiple ISVs—which are commonly found in natural mosquito populations—might impose cumulative barriers to arbovirus dissemination through complementary mechanisms, such as targeting different stages of the viral life cycle or activating distinct immune pathways. Indeed, Olmo et al. [26] demonstrated that co-infection with PCLV and HTV enhances dengue and Zika virus transmission in *Ae. aegypti*, highlighting the bidirectional nature of ISV-arbovirus interactions. Future research should systematically screen pairwise and multi-ISV combinations in both cell lines and mosquito models to identify synergistic pairs, with priority given to those that naturally co-occur in field populations. If successful, such multi-ISV strategies could be integrated into vector control programs, either by establishing stable infections in laboratory-reared mosquitoes prior to field release or by leveraging their VT to spread through wild populations. However, the safety and ecological impact of releasing ISV-infected mosquitoes would require careful assessment, particularly regarding the potential for unintended interactions with other pathogens or non-target organisms.

## Figures and Tables

**Table 1:** Sequences of primers and probes used in this study.

Primers and Probes	Sequences (5′-3′)
CFAV-F	ACACGAGTGAAGCTGGTTGA
CFAV-R	ACATACGTTCCTGGTTCCCG
CFAV-P	FAM-CCCGTCCTCCCTCTCCTCTGGATC-BHQ1
ZIKV-F	AAGTTTGCATGCTCCAAGAAAAT
ZIKV-R	CAGCATTATCCGGTACTCCAGAT
ZIKV-P	FAM-ACCGGGAAGAGCATCCAGCCAGA-BHQ1
actin-F	TCCCACACAGTCCCCATCTA
actin-R	ACGAGTAGCCACGTTCAGTCAG
actin-P	VIC-CGCTCGCGATCTGACCGATTATCTGAT-BHQ1
rps6-F	CGTCGTCAGGAACGTATCC
rps6-R	TTCTTGGCAGCCTTAGCAG
rps6-P	VIC-CGTCTGTCCTCGATGCGTGA-BHQ1

**Table 2:** Infection rate of CFAV in the progeny from different GCs of *Ae. albopictus*.

Gender	GC1	GC2	GC3
Female	0/20	0/20	0/8
Male	0/20	0/20	0/9

## Data Availability

The data presented in this study are available on request from the corresponding authors.

## Abbreviations

The following abbreviations are used in this manuscript:

ISV	Insect-specific viruses
CFAV	Cell-fusing agent virus
ZIKV	Zika virus
PBS	Phosphate-buffered saline
dpi	days post-infection
GC	gonotrophic cycle
VT	vertical transmission
VTR	vertical transmission rate
